# Communication as Mediator: Interprofessional Deprescribing and the RCC (Roles–Collaboration–Communication) Framework

**DOI:** 10.3390/healthcare14172739

**Published:** 2026-08-27

**Authors:** Alina Cernasev, Devin Scott, Laura Reed, Amy Hall, Bruce L. Keisling

**Affiliations:** 1 Department of Clinical Pharmacy and Translational Science, College of Pharmacy, The University of Tennessee Health Science Center, Nashville, TN 37211, USA; 2 Department of Interprofessional Education, College of Graduate Health Sciences, The University of Tennessee Health Science Center, Memphis, TN 38163, USA; 3 Department of Health Promotion and Disease Prevention, College of Nursing, The University of Tennessee Health Science Center, Memphis, TN 38163, USA; 4 University of Tennessee Health Science Center, Memphis, TN 38163, USA; 5 Departments of Pediatrics and Medical Education, College of Medicine, The University of Tennessee Health Science Center, Memphis, TN 38163, USA; bkeislin@uthsc.edu

**Keywords:** deprescribing, healthcare professional students: polypharmacy, interprofessional education

## Abstract

**Highlights:**

**What are the main findings?**

- The Roles–Collaboration–Communication (RCC) interprofessional framework illustrates how interprofessional healthcare teams use communication to navigate clearly defined roles and facilitate collaboration in complex processes such as deprescribing.
- By applying this framework, healthcare professionals can enhance teamwork and patient care, particularly in complex processes such as deprescribing.

**What are the implications of the main findings?**

- An interprofessional approach to deprescribing, where doctors, pharmacists, nurses, and other healthcare professionals collaborate, is necessary to ensure comprehensive medication reviews, shared decision-making, and safer, more effective patient care.
- Understanding polypharmacy and practicing deprescribing within an interprofessional team through an unfolding process prepares healthcare students to address complex medication regimens, prioritize patient safety, and promote optimal therapeutic outcomes in their future clinical practice.

**Abstract:**

**Background:** Polypharmacy remains a significant challenge, often leading to adverse drug events and increased healthcare costs. One effective strategy to mitigate these risks is deprescribing, the systematic and intentional discontinuation or reduction of medications. Despite growing recognition of deprescribing’s importance, limited research has examined the impact of interprofessional education interventions on developing deprescribing competencies among healthcare professional students. This study aims to examine the perspectives of healthcare students, including student pharmacists, medical students, and nurse practitioner students, regarding their roles and responsibilities in the deprescribing process. **Methods:** Following completion of a Deprescribing Interprofessional Education Simulation activity (DIPE-SA), participants were invited to join focus groups. The study included students from the University of Tennessee Health Science Center (UTHSC) Colleges of Medicine, Nursing, and Pharmacy. Constructivist Grounded Theory (CGT) underpinned the qualitative research approach, informing the development of the study framework. Each focus group was facilitated by two researchers and recorded. Recruitment continued until saturation was achieved. Data were transcribed verbatim and analyzed for themes using Dedoose, qualitative analysis software. **Results:** A total of 19 participants attended four focus groups. Three major themes emerged and guided the development of the Roles–Collaboration–Communication (RCC) interprofessional framework. The first theme, *roles*, focuses on the unique responsibilities and functions healthcare professionals assume within the team and throughout the deprescribing process. The second theme, *collaboration*, emphasizes coordinated efforts and shared decision-making among members of the interprofessional healthcare team in deprescribing. Finally, the third theme, *communication*, highlights the importance of effective information and intention exchange, serving as both a mediator and connector, linking roles and collaboration, and ultimately enabling successful deprescribing. **Conclusions:** This study’s findings, together with the RCC interprofessional framework, demonstrate the interplay of roles, collaboration, and communication in medication management. The RCC framework highlights the value of dynamic team processes for achieving safe and effective deprescribing, which in turn improves patient care and health outcomes.

## 1. Introduction

Polypharmacy is a continuously growing concern in the care of older adults, contributing significantly to adverse drug events, hospitalizations, and decreased quality of life. As the aging population continues to expand, so does the number of individuals managing complex medication regimens, often without adequate oversight to ensure continued clinical appropriateness. Deprescribing is a structured process that reduces or discontinues medications where the harm outweighs the benefits and offers a promising approach to mitigating medication-related risks and aligning treatment with patient goals of care [1,2]. Evidence shows that deprescribing can reduce inappropriate medication use without worsening symptoms, thereby improving patient safety and quality of life [3,4].

Despite these benefits, deprescribing remains underutilized in clinical practice, in part due to gaps in professional training. Educational exposure to deprescribing practices during pharmacy school, for example, is inconsistent, and many student pharmacists report limited confidence and report feeling unprepared to implement deprescribing strategies in real-world settings [1]. Similar gaps have been identified across medical and nursing education programs, raising concern that current health professional curricula may not adequately equip future clinicians to address polypharmacy through a collaborative, interprofessional lens [4,5]. While some postgraduate programs have incorporated deprescribing training into residency experiences, graduate education still lacks comprehensive, integrated instruction on this critical aspect of geriatric care [2].

Interprofessional education (IPE) experiences present a unique opportunity to strengthen deprescribing competence among emerging healthcare providers. Programs such as the Initiative to Minimize Pharmaceutical Risk in Older Veterans (IMPROVE) have demonstrated that team-based deprescribing education enhances knowledge and fosters greater appreciation for the roles of other disciplines involved in medication management [3]. Student pharmacists bring unique expertise in pharmacotherapy and medication review to IPE experiences, positioning them to lead or support deprescribing efforts when adequately trained and empowered through interprofessional collaboration. Their evolving role highlights the importance of collaborative practice models that leverage the strengths of each profession in optimizing patient care [3].

Effective interprofessional collaboration in healthcare is essential for delivering high-quality, patient-centered care. Recognizing the limitations of traditional models that emphasize only individual professional competencies, McLaney et al. (2022) introduce a team-based framework that shapes how healthcare teams work together [6]. Developed through extensive literature review and stakeholder consultation, this model identifies six core competencies crucial to collaborative practice: communication, conflict resolution, shared decision-making, reflection, role clarification, and interprofessional values [6]. These competencies are actionable and collective, offering a shared language for both clinical and non-clinical staff to enhance care delivery. By integrating the framework into hospital operations, including interactive staff training, new employee orientation, and organizational evaluation tools, the initiative fosters a culture of collective competence [6]. In this context, student pharmacists, medical students, and nurse practitioner students can be equipped with the necessary skills to contribute meaningfully to team-based care [6]. Ultimately, this approach aims to improve health system outcomes and team performance in complex hospital settings, highlighting that optimal care results from the integrated efforts of the whole team, including patients and families [6].

Despite these advancements, few studies have examined how interprofessional education interventions can specifically build deprescribing skills among healthcare professional students, or how these efforts may reduce perceived barriers to deprescribing. This highlights a critical opportunity to embed deprescribing into interprofessional learning environments to prepare future healthcare professionals to improve in this area and ensure that deprescribing becomes a routine part of patient-centered care.

Prior research revealed a knowledge gap among student pharmacists regarding how to initiate and operationalize deprescribing in practice [7]. Addressing this gap is critical to ensuring that future healthcare professionals are well-prepared to embrace deprescribing as a routine aspect of patient care, benefiting from the foundation of collaborative competencies emphasized in modern IPE models.

This study seeks to better understand how healthcare students, specifically student pharmacists, medical students, and nurse practitioner students, view their roles in the deprescribing process. By exploring their thoughts and experiences following a shared Deprescribing Interprofessional Education Simulation Activity (DIPE-SA), we aim to identify strategies to help healthcare training overcome existing barriers to deprescribing.

## 2. Materials and Methods

Students from the Colleges of Pharmacy, Medicine, and Nursing at the University of Tennessee Health Science Center (UTHSC) were recruited to participate in a voluntary, ungraded Deprescribing Interprofessional Education Simulation Activity (DIPE-SA) [8]. The methods consisted of two main components. First, students engaged in a virtual DIPE-SA event based on an unfolding case scenario designed to teach deprescribing practices. This simulation presented a complex patient with polypharmacy issues and included prebriefing, the activity itself, and debriefing [8].

The case evolved over multiple stages, with new information and challenges introduced at each step to mimic real-world complexity [8]. At the conclusion of the DIPE-SA, participants were invited to voluntarily join focus groups to provide feedback and discuss their experiences. These qualitative insights were integral to understanding the educational impact of the DIPE-SA. Participants received gift cards for their time. Further methodological details about the DIPE-SA are available in the study by Reed et al., 2026 [8].

The focus group discussion methodological approach was chosen because it effectively collects data on complex concepts and activities [9]. Additionally, focus groups encourage diverse perspectives and help researchers identify emerging themes from participants. Through these discussions, researchers can better understand participants’ perceptions, motivations, and experiences [9].

Participants attended a DIPE-SA session and selected their preferred days and times to participate in focus groups held via Zoom^®^. IRB approval was obtained from the institution. Trained moderators with PhDs (AC, DS) and extensive expertise in qualitative research led all focus groups. DS is not currently teaching in any of the professional colleges, and AC is currently teaching in the College of Pharmacy, minimizing potential bias in the discussions [10]. Sessions continued until data saturation was reached in the fourth focus group in Spring 2025, wherein question responses were predictable and reinforced previously collected data without providing new insights [10,11].

Participants were asked open-ended questions to gather their perspectives on deprescribing during the DIPE-SA, identify potential improvements to the activity, and understand the roles of each profession involved. The discussion guides were reviewed and refined after each focus group session to enhance clarity and incorporate new discussion topics for subsequent sessions. Each focus group lasted 60–75 min and was conducted via Zoom^®^. To maintain objectivity, all sessions were video- and audio-recorded, and a third party transcribed the audio recordings verbatim [10]. More information can be found in Supplementary Materials [10].

The transcripts were imported into Dedoose^®^ (Version 9.0.17, Los Angeles, CA, USA) to organize and manage all qualitative data. Two researchers (AC, AH) with prior experience and training in qualitative research conducted the analyses and validated the findings. Both researchers coded all transcripts, and, when inconsistencies arose, the team (DS, BK) discussed and resolved them. Coding definitions, team decisions, and emerging themes were documented in an audit trail to ensure analytical rigor [12].

A semi-structured focus group guide was specifically designed for each group using a “funnel” approach, which allows for progression from broader topics to more specific issues. This method helped ensure that all relevant aspects related to the DIPE-SA activity were thoroughly covered [9].

Constructivist Grounded Theory (CGT) was chosen as the foundation for qualitative research [12]. In practice, CGT was applied through several key steps: initial coding of interview transcripts, the use of constant comparison to refine codes, and memo-writing to capture analytic insights throughout the process. Categories were developed iteratively as patterns emerged from the data, with ongoing memo-writing supporting the integration of concepts [12]. This systematic approach facilitated the progression from initial codes to broader categories and ultimately to the three final themes [12]. This methodology aims to explore how students navigate the development of their professional identity and adapt to clinical decision-making in interprofessional environments. By employing a CGT approach, we deepen our understanding of the student learning experience and identify systemic or environmental factors that influence professional growth. In the context of DIPE-SA, CGT promotes critical reflection, encourages evidence-based practice, and contributes to educational research aimed at enhancing experiential learning and curriculum development [12].

Unlike the traditional grounded theory approach developed by Glaser and Strauss, which adopts a more objectivist stance, Charmaz’s constructivist version recognizes the influence of the researcher’s perspective and the social context on the data and the emerging theory [12]. For example, in CGT, data collection and analysis happen simultaneously. Researchers engage in open, focused, and theoretical coding while writing memos to capture insights and develop concepts [12].

## 3. Results

A total of 52 students from three colleges, representing four campuses in the mid-southern United States, participated in the DIPE-SA. Of these, 21 were MD students from the College of Medicine, 20 were PharmD (Doctor of Pharmacy) students from the College of Pharmacy, and 11 were DNP (Doctor of Nursing Practice) and FNP (Family Nurse Practitioner) students from the College of Nursing. Nineteen participants took part in four focus group interviews. Medicine and pharmacy students attended all four sessions, while nurse practitioner students participated in three. More details can be found in Table 1.

**Figure 1:**
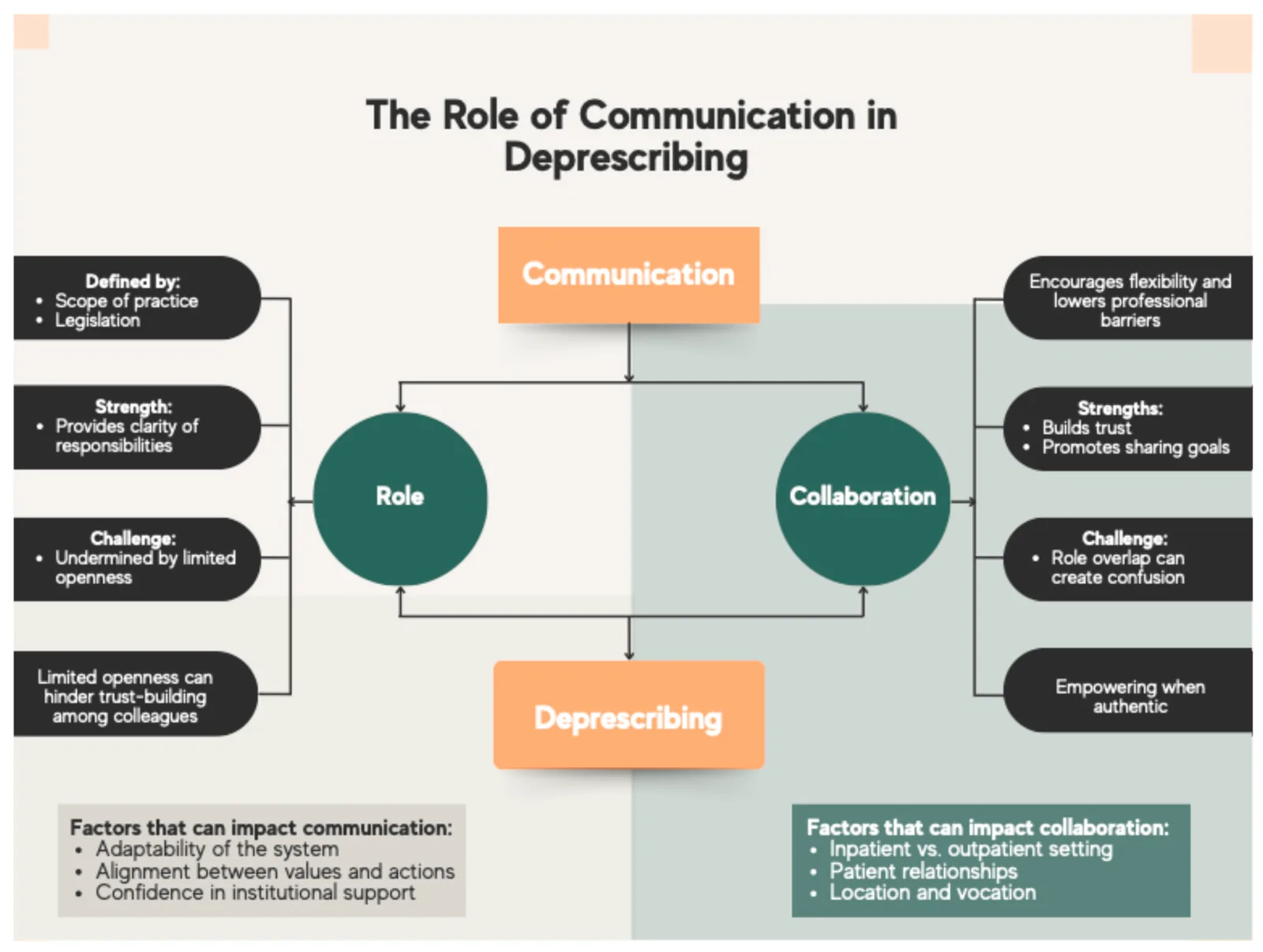
Roles–Collaboration–Communication (RCC) interprofessional framework.

Three major themes emerged and guided the development of the Roles–Collaboration–Communication (RCC) interprofessional framework, as depicted in Figure 1. The RCC framework offers a valuable perspective for understanding and optimizing interprofessional collaboration. Deprescribing, which involves systematically identifying and withdrawing medications that may no longer be necessary or that may be causing harm, requires concerted healthcare team efforts from diverse healthcare professionals, including physicians, pharmacists, nurses, and others. Each team member brings their own expertise and responsibilities, but effective deprescribing depends on transcending rigid professional boundaries and working collaboratively toward patient-centered outcomes.

The first theme, *roles*, centers on the roles healthcare professionals play in the healthcare team and in the deprescribing process. The second theme, *collaboration*, refers to efforts to work together among members of the interprofessional healthcare team, particularly in deprescribing. Finally, the third theme, *communication*, highlights communicative acts that serve as mediators or connectors, bridging the disparate themes of role and collaboration to facilitate successful deprescribing. Table 2 presents the themes with a representative quotation.

**Theme 1: Role: *“I thought that [the activity] highlighted the different roles that we have on the healthcare team.”*** (S2, FG1, Pharmacy)

Focus group participants highlighted what they saw as well-defined roles for members of the healthcare team. A student pharmacist indicated that “*we each had different directives in the activity to lead the discussion, and I thought that highlighted the different roles that we have on the healthcare team*,” (S2, FG1, Pharmacy), with a medicine student concurring: *“I agree with [her].”* (S1, FG1, Medicine) Similarly, one student noted that the activity “helped, at least from a medicine perspective, see where our gaps are and where pharmacy has more knowledge.” (S4 FG1 Medicine) Finally, the exchange was closed by another student, stating, “I agree with all of them.” (S3, FG1, Pharmacy)

Participants went on to define the role of prescriber as the initiator of deprescribing while defining the pharmacist as the medication expert. One stated: “I would say the initiator of deprescribing is a physician.” (S9, FG3, Medicine) Another agreed, responding “I would say [physicians are] both an initiator and a centralized point of contact, potentially, for other disciplines.” (S12, FG3, Medicine) Still another agreed: “Yeah, I kind of agree with [(S12, FG3, Medicine)] and [(S9, FG3, Medicine)], I guess physicians are expected to be initiators, I think a lot of times, I mean like the leaders of the healthcare team.” (S11, FG3, Medicine) A nurse practitioner student expanded the role of initiator of deprescribing to nurse practitioners, stating, “I think so, either the NP or the MD, because we’re going to be the ones that have the face-to-face contact.” (S8, FG2, Nursing)

Similarly, participants saw the role of a pharmacist as a medication expert. A student pharmacist stated that physicians and nurse practitioners were responsible for initiating deprescribing, while pharmacists make recommendations about discontinuing therapies: “The pharmacist on the team is equipped very well to find those duplicate therapies, inappropriate therapies, and to make the recommendations to discontinue some of them, and that MDs or NPs would generally be the ones that would be initiating the deprescribing.” (S6, FG2, Pharmacy)

Another participant offered a similar viewpoint grounded in her experience in the interprofessional deprescribing simulation: “I agree with [(S7, FG2, Medicine)]. When I went in, I was like, oh, the pharmacist is going to know all these meds. They’re going to know exactly what to take away, and they really did.” (S8, FG2, Nursing) Student pharmacist participants agreed that the pharmacist’s role in deprescribing is the medication expert, with (S18, FG4, Pharmacy) describing the pharmacist: “Yeah, I completely agree with (S19, FG4, Pharmacy), just like making sure the medication-- like just having the medication list, going through it, getting rid of all the duplicates, checking to see if there are any drug interactions, checking to see if the patient even needs to be on it, and checking if the dosing is correct.”

**Theme 2: Collaboration: *“Deprescribing is a team effort.”*** (S5, FG2, Pharmacy)

Another emergent theme was collaboration. Participants indicated the importance of collaboration among an interprofessional healthcare team and called attention to the benefits of collaboration for deprescribing. A participant asserted the collaborative aspect of deprescribing, stating: “I would definitely say that everybody on the healthcare team is involved in the deprescribing process. Everybody has their own unique role to play, but everybody is involved in looking at what’s appropriate and what’s not appropriate.” (S6, FG2, Pharmacy)

Another participant agreed, saying: “I was going to say the exact same thing, deprescribing is a team effort… different information resonates with each different role.” (S5, FG2, Pharmacy)

Likewise, a nurse practitioner student summarized: “It’s good to know that we have different specialties. We have other people that we can count on. And it’s good to rely on pharmacy and our specialists for information.” (S16, FG4, Nursing)

While highlighting the leadership role of physicians on the healthcare team, (S11, FG3, Medicine) attributes successful deprescribing to collaboration: “So I think being an initiator is kind of an expectation for [physicians] but also being that central point contact, someone who is willing to reach out to the other disciplines and collaborate with them to come to a best conclusion on how to move forward with a patient.” Likewise, a medicine student participant brings attention to collaboration when deprescribing: “on a lot of the teams that I’ve worked with inpatient, we’ve had a pharmacist working on the team and I’ve always really appreciated their knowledge and just what they know about all of these medications and how they’re able to really support us… so I would say that this experience continued to affirm that belief that just pharmacists are incredibly useful, beneficial individuals who are able to really contribute to the care that we’re able to provide for our patients.” (S7, FG2, Medicine)

Here, this participant indicates that pharmacists play a crucial collaborative role in the interprofessional deprescribing team. Another participant speaks to the benefits of collaboration between prescribers and pharmacists: “Every time I interact with pharmacy students or pharmacists, I’m always knowing that they’re going to be super, super helpful for filling in my knowledge gaps. And every time I’m always impressed even more by how much they know and how much they can help me.” (S4 FG1 Medicine) Another student concurred, stating, “My takeaway would be that it’s always important for me to work really closely with and, you know, get the opinions of pharmacy… when it comes to deprescribing.” (S1, FG1, Medicine) They go on to state: “the takeaway would be to always try to keep them involved in the conversations and get their medical advice.”

**Theme 3: Communication: “*To do deprescribing… I need to understand the areas of expertise with my colleagues and be comfortable communicating with and reaching out to them.”*** (S12, FG3, Medicine)

Theme three centers on communication and includes two subthemes: 1) communication as challenge and 2) communication as mediator. In theme three, participants highlight communication as a concern when deprescribing. That said, they also indicate that communication is the mediator that brings healthcare professionals with distinct roles together to collaborate as an interprofessional healthcare team.

**Subtheme 3.1: Communication as Challenge:** “I’m worried that there could be additional confusion as a result of a patient having multiple different providers.” (S7, FG2, Medicine)

One participant indicated that they had seen a different level of communication between members of the healthcare team in terms of deprescribing in the clinic and hospital setting: “in the clinic, I just really haven’t, in my experience, like seen the NP call the pharmacist… But in the hospital, it’s the opposite. You know, like we do rounds together and they tweak things here and there.” (S13, FG3, Nursing)

A student pharmacist agreed and highlighted the need to build rapport between team members: “Again, it all kind of comes from your practice setting… I think the biggest thing to overcome is building that rapport with my provider… Knowing that they trust me as a supporting provider as well when I do make those recommendations.” (S5, FG2, Pharmacy)

In terms of building rapport, (S15, FG4, Medicine) indicated that communication medium can present a challenge to successful deprescribing: “Sometimes you’re communicating over a secure chat and you’re waiting for people to respond, so that could lead to issues with communication. And especially if-- maybe if you haven’t met the person before, haven’t worked with them a lot, that would be harder to communicate.”

Here, participants discussed the different communication dynamics in a hospital versus a clinical setting, highlighting the lack of interprofessional communication among healthcare teams in a clinical setting.

Several participants spoke about their concerns surrounding initial and follow-up communication for deprescribing. A student pharmacist stated: “In primary care, I’m not sure who to go to, to deprescribe… I’m actually not sure would I go to the physician or would I go to the mid-level first.” (S2, FG1, Pharmacy) Another participant echoed the student pharmacist’s concern about initial contact, stating: “Following up on what [she] just said, I think if-- I know this happens a lot, but when multiple providers have prescribed the-- basically like the same medication almost… I also don’t know who first to go to.” (S3, FG1, Pharmacy)

A medicine student continued this discussion of communication concerns when there are multiple providers, saying “I’m worried that there could be additional confusion as a result of a patient having multiple different providers.” (S7, FG2, Medicine) Finally, a nurse practitioner student echoed that concern: “That’s also a concern for us in the outpatient. You know, A, if they get re-hospitalized, are they going to be put back on something? And, B, like aside from that, when they go home, are they actually going to pay attention and discontinue the correct med or are they going to discontinue the wrong one and be in a worse spot or not listen at all.” (S8, FG2, Nursing)

Here, participants highlighted the communication difficulties faced by members of the interprofessional healthcare team.

**Subtheme 3.2: Communication as Mediator:** “In order to do deprescribing… I need to understand the areas of expertise with my colleagues and be comfortable communicating with and reaching out to them.” (S12, FG3, Medicine)

In addition to highlighting communication difficulties, participants called attention to communication as a mediator that links healthcare professionals with disparate roles into a collaborative interprofessional healthcare team. One medicine student asserted that “In order to do deprescribing… I need to understand the areas of expertise with my colleagues and be comfortable communicating with and reaching out to them.” (S12, FG3, Medicine)

Another participant agreed, stating: “Deprescribing is a collaborative effort and that we have a bunch of different resources and a bunch of different individuals who are able to bring different perspectives in order to contribute to helping a patient deprescribe.” (S7, FG2, Medicine)

A student pharmacist also echoed the need for effective communication to deprescribe, stating that “working towards being a good initiator with effective communication is key to supplying our patients with the best care.” (S14, FG3, Pharmacy) A nurse practitioner student offered a similar takeaway: “Yes, I agree, just collaboration with your colleagues and asking plenty of questions in order to deprescribe.” (S13, FG3, Nursing) Another participant concludes: “Yeah, I would agree. I think communication is one of the biggest takeaways of this activity, kind of being able to see what your responsibilities are and what you can kind of delegate or reach out to other disciplines for to get the best recommendations and the best approach on how to treat a patient.” (S11, FG3, Medicine)

Participants repeatedly emphasized the importance of communication for successful deprescribing, describing it as a mediator that brings disparate roles together in a collaborative effort.

Participants also set improved communication as a deprescribing goal. One participant said: “I think one of the things that I could personally improve on would be my own communication with other providers, being able to communicate effectively and efficiently what it is that I want to change or deprescribe, why I want to do it, and doing that in a consolidated amount of time to where the point gets across and they agree with me.” (S6, FG2, Pharmacy)

Another participant made a similar point, stating: “The overall lesson I learned is that deprescribing isn’t always going to be successful, but that doesn’t mean that you can’t continue a conversation and come back to it at the next appointment.” (S3, FG1, Pharmacy) Ultimately, communication has the capacity to serve as a mediator bridging roles and collaboration, but a lack of communication can serve to hinder deprescribing by reinforcing distinct roles and preventing collaboration.

## 4. Discussion

Three major themes emerged and guided the development of the Roles–Collaboration–Communication (RCC) interprofessional framework. This study provides insights into the deprescribing process by illustrating how communication acts as a mediator linking the distinct roles of healthcare professions with the need for interprofessional collaboration, as illustrated in Figure 1.

Previously published deprescribing frameworks, such as Linsky et al., 2019 [13], present a comprehensive structure that integrates diverse influences and moves beyond the narrow focus of earlier tools. While those frameworks frequently focused on particular medication classes or discrete clinical steps, their models organize the field broadly to help prioritize research and design interventions [14,15]. In contrast, our RCC interprofessional framework offers a unique perspective that invites further discussion. Specifically, it uncovers important differences in how professional roles are conceptualized within the healthcare team during deprescribing. The RCC framework explicitly maps out the responsibilities and interactions of each discipline, making visible the dynamic processes that underpin successful deprescribing initiatives. Where previous frameworks [13,15] may oversimplify or understate the nuances of teamwork, the RCC framework highlights the necessity of interprofessional collaboration and positions communication not just as a facilitator, but also as the central mediator for change (Figure 1). By comparing the RCC framework with prior models, we gain new insights into the mechanisms that drive interprofessional deprescribing and can better identify opportunities for improvement.

Furthermore, Almodovar et al., 2024 [16], introduce a socioecological (SEM) framework to improve deprescribing, emphasizing the multifaceted challenges encountered in clinical practice. The authors highlight how the involvement of multiple prescribers can lead to fragmented communication, thereby increasing risks for patients with complex health needs [16]. The SEM framework systematically considers various factors at the individual, interpersonal, organizational, and societal levels that shape deprescribing efforts [16]. Notably, the framework positions communication as a primary obstacle for prescribers [16]. However, our findings suggest a more nuanced role for communication in the deprescribing process. While communication can indeed act as a barrier, it can also serve as a facilitator, catalyzing effective collaboration and supporting shared decision-making among healthcare providers. This duality highlights the importance of examining the quality and context of communication, rather than simply categorizing it as a barrier, to advance deprescribing initiatives.

Kassis et al., 2024 [17], provide valuable insights into how different stakeholders perceive the pharmacist’s role in reducing inappropriate medication use. Their synthesis of nine qualitative articles from five countries identified four overarching themes related to pharmacist roles in ambulatory settings [17]. Notably, the theme of “Role and Responsibility” reveals considerable uncertainty about which healthcare professional should initiate deprescribing interventions [17]. This ambiguity is not only evident in qualitative meta-synthesis but also mirrors our own findings, particularly in relation to healthcare professional students’ perspectives [17]. The theme from the qualitative meta-synthesis emphasizes the pharmacists’ perceived need for the “imprimatur of prescribers,” which represents a formal approval from a physician and highlights the persistent concerns about professional boundaries and interprofessional collaboration [17].

Taken together, the qualitative meta-analysis findings and our qualitative data highlight ongoing challenges in defining and operationalizing the pharmacist’s responsibilities in deprescribing practices, suggesting a need for clearer interprofessional protocols and educational initiatives to empower each provider within multidisciplinary teams. Building on this, recent research has explored practical strategies to enhance interprofessional collaboration and clarify these roles in educational settings.

For example, Haddad et al., 2025, investigate the impact of virtual simulations with standardized patients on enhancing interprofessional collaboration and ethical decision-making among student pharmacist and nurse practitioner students [18]. In their study, students work in pairs to navigate challenging clinical scenarios, followed by in-depth debriefing sessions that encourage critical reflection on their actions and decisions [18]. Key themes emerging from these discussions include the importance of patient-centered care, the cultivation of mutual trust among different healthcare professionals, and the role of joint problem-solving [18]. Although these synchronous, web-based simulations are resource-intensive, the results indicate that they effectively equip students to meet accreditation standards expected in professional practice [18]. Ultimately, the study highlights the pivotal role of reflective dialogue in moving healthcare students beyond basic information exchange toward genuine, integrated ethical teamwork, aligning with the need for more cohesive interprofessional approaches highlighted earlier [18].

In contrast, the physician’s primary role in deprescribing centers on clinical decision-making authority, diagnostic integration, and accountability for initiating, modifying, or discontinuing medications within the broader context of the patient’s overall healthcare plan [19]. Physicians are uniquely positioned to evaluate the ongoing indication, risk–benefit profile, and alignment of each medication with the patient’s current goals of care while also managing potential withdrawal effects and monitoring clinical outcomes following deprescribing [19]. Importantly, physicians play a critical coordinating role by legitimizing deprescribing recommendations within interprofessional teams and engaging in shared decision-making with colleagues and patients, thereby helping to overcome therapeutic inertia and ensuring that deprescribing efforts are safe, patient-centered, and integrated into routine care [19].

Nurse practitioners emerged in our findings as both complementary to and overlapping with physician colleagues in the deprescribing process. Like physicians, nurse practitioners act as key initiators of deprescribing, demonstrating their prescriptive authority and direct patient engagement, particularly in outpatient settings. Their role within the interprofessional team represents a vital intersection of clinical decision-making, patient education, and continuity of care. Almodovar et al [16]. highlight that effective communication is central to the nurse practitioner’s impact. Participants noted uncertainty regarding whom to contact when multiple prescribers are involved, apprehension about medication adjustments during care transitions, and risks associated with patients misunderstanding deprescribing instructions [16]. Our findings reinforce that communication serves not only as a potential barrier but also as a critical facilitator, supporting multidisciplinary teams in fostering safe and collaborative deprescribing practices throughout the care continuum.

Hawkins et al., 2021 [20], conducted a qualitative study that explored the barriers and facilitators experienced by healthcare providers when deprescribing concurrent opioids and benzodiazepines within the Veterans Affairs system. Their findings revealed that challenges such as patient resistance and limited consultation time significantly hinder safe discontinuation [20]. These obstacles are commonly encountered in clinical practice [20]. Notably, the study also emphasized the unique position of pharmacists, who are well-equipped to support tapering efforts through medication reviews, patient education, and close collaboration with both primary care and mental health providers [20]. As we reflected on Hawkins et al.’s 2021 findings [20], we recognized important parallels with our own findings regarding the roles of each team member, particularly the essential contributions pharmacists make within the interprofessional team.

Our findings from focus group discussions following this DIPE-SA reinforce the critical importance of interprofessional collaboration in successful deprescribing initiatives. Each member of the healthcare team, including physicians, pharmacists, nurses, and others, brings a unique perspective and set of skills that contribute to comprehensive patient care. Clark et al., 2020 [21], demonstrated the impact of a pharmacist-led pilot program aimed at reducing potentially inappropriate medications among elderly patients during Medicare wellness visits. In this program, a clinical pharmacist played a pivotal role in identifying polypharmacy risks and making tailored deprescribing recommendations to physicians [21]. However, the overall acceptance rate for these recommendations was only 20%, highlighting the persistent hesitation among both patients and providers to modify established drug regimens [21]. Several obstacles hindered deprescribing implementation, including limited access to electronic health records and the absence of well-established interdisciplinary relationships [21]. These challenges were echoed in our focus group discussions, which revealed that while interprofessional team collaboration was valued, it was often constrained by organizational and logistical issues, including rigid professional roles. To overcome these obstacles, integrating clinical workflows, fostering strong interdisciplinary relationships, and open communication are essential. By leveraging each team member’s unique expertise and promoting effective communication, interprofessional teams can optimize the deprescribing process and ultimately improve patient outcomes.

A critical examination of existing interprofessional team collaboration models reveals significant limitations in their ability to capture the nuanced realities of deprescribing practices. For instance, the framework of McLaney et al., 2022, while influential, does not fully elucidate the intricate interplay of roles, communication, and collaboration inherent to effective deprescribing [6]. Although the framework of McLaney et al., 2022, identifies Role Clarification, Shared Decision Making, and Communication as three of its six core competencies, it falls short in addressing the persistent tension between rigidly defined healthcare roles and the aspiration for seamless collaboration [6]. Notably, Communication is presented as a static competency, a skill to be acquired, rather than as a dynamic and mediating force [6]. As a result, the framework does not fully account for the ways in which communication actively bridges or reinforces boundaries between roles, limiting its capacity to foster authentic interprofessional teamwork [6].

Brewer et al., 2012, introduce Curtin University’s Interprofessional Capability Framework, a comprehensive pedagogical model aimed at equipping students with the skills necessary for effective collaboration in the health workforce [22]. This innovative framework integrates three core pillars: client-centered service, safety and quality, and collaborative practice [22]. These pillars serve as the foundation for the curriculum, which features clearly defined levels of achievement, allowing for the systematic assessment of student development from novice to entry-level professional across multiple disciplines [22]. Central to the program are five essential capabilities: communication, reflection, role clarification, conflict resolution, and team function [22]. However, while communication is recognized as a core capability alongside role clarification, the Brewer framework, like McLaney’s, does not conceptualize communication as a mediating process between roles and collaboration [6,22]. Instead, it is treated as one discrete skill among several, rather than as the mechanism by which boundaries are negotiated and collaboration is enacted.

In contrast to these prevailing models, this manuscript introduces the RCC interprofessional framework, which is explicitly designed to foreground the tension between professional roles and collaborative intent. The RCC framework posits that effective deprescribing hinges not only on clarifying roles and fostering collaboration, but also on recognizing communication as an active mediator that can either facilitate or impede the transition from hierarchical roles to authentic teamwork. Unlike the McLaney and Brewer frameworks, which treat communication as a static competency or one capability among many, the RCC framework positions communication as the dynamic mechanism that shapes, negotiates, and transforms interprofessional relationships [6,22]. This perspective opens a discussion regarding the limitations of current educational approaches, arguing for a pedagogical shift that explicitly prioritizes communication skills and strategies aimed at navigating and overcoming entrenched professional boundaries. By reframing communication as a central, mediating process, the RCC framework offers a more comprehensive and realistic lens for understanding and teaching interprofessional deprescribing.

### Strengths and Limitations

Utilizing CGT as a methodological approach provided a robust foundation for exploring complex social processes within healthcare education, such as deprescribing as an interprofessional team. CGT, known for its reflexive and iterative nature, enabled the researchers to uncover nuanced insights into how interprofessional teams approach deprescribing. The qualitative study design and the use of CGT facilitated the development of the RCC framework to capture a collective perspective on the interprofessional team’s thinking on deprescribing initiation. This framework could be used by researchers across healthcare when working as an interprofessional team. Furthermore, the online platform allowed medical students, nurse practitioner students, and student pharmacists from the same university, located in different parts of the state, some of whom were enrolled in their final-year practical rotations, to participate in the activity and share their views on deprescribing. However, the recruitment and sample characteristics may limit the generalizability of the findings to other U.S. health science institutions or clinical practice. Since all participants were recruited from a single institution, this limitation should be emphasized, as it restricts the extent to which the results can be applied to broader settings. The findings from the current study highlight the need for more longitudinal studies to explore deprescribing as an interprofessional team across the curricula of other healthcare programs.

## 5. Conclusions

This study’s findings, together with the RCC interprofessional framework, demonstrate that clear role delineation ensures accountability in medication management. Collaboration enables the team to combine their knowledge and skills to address complex clinical decisions. Most importantly, open and ongoing communication facilitates the sharing of information, addresses concerns, and builds consensus around deprescribing plans. The RCC framework thus highlights the value of dynamic team processes for achieving safe and effective deprescribing, which in turn has the potential to improve patient care and health outcomes. By recognizing and adopting these elements, these findings suggest that healthcare professionals can collaborate more effectively to achieve shared goals, such as deprescribing, ultimately fostering a more cohesive team environment and enhancing patient outcomes.

To further maximize the impact of the RCC interprofessional framework, practical recommendations should be considered for integrating it into healthcare curricula and future interprofessional training programs. Specific strategies may include developing targeted educational modules, incorporating scenario-based learning, and facilitating interdisciplinary workshops that emphasize the application of the RCC principles in real-world clinical settings. Continued evaluation and refinement of the framework in varied clinical environments will be important to ensure its generalizability and effectiveness. Exploring the impact of the RCC framework on patient outcomes, interprofessional collaboration, and implementation barriers in diverse contexts could provide valuable insights for ongoing improvement.

## Figures and Tables

**Table 1:** Demographics of Student Participants.

College	Number of Participants
College of Medicine	8
College of Nursing	3
College of Pharmacy	8
**Graduation Year**	
**2025**	16
College of Medicine	8
College of Nursing	3
College of Pharmacy	5
**2026**	3
College of Medicine	0
College of Nursing	0
College of Pharmacy	3

**Table 2:** Themes with Representative Quotes.

**Theme 1**	**Role** **: *“I thought that [the activity] highlighted the different roles that we have on the healthcare team”* (S2, FG1, Pharmacy)**	
**Theme 2**	**Collaboration**: *“Deprescribing is a team effort.”* (S5, FG2, Pharmacy)	
**Theme 3**	**Communication**: “*To do deprescribing… I need to understand the areas of expertise with my colleagues and be comfortable communicating with and reaching out to them.”* (S12, FG3, Medicine)	
**Subthemes**	3.1: **Communication as Challenge:** “I’m worried that there could be additional confusion as a result of a patient having multiple different providers.” (S7, FG2, Medicine)	3.2: **Communication as Mediator:** “In order to do deprescribing… I need to understand the areas of expertise with my colleagues and be comfortable communicating with and reaching out to them.” (S12, FG3, Medicine)

## Data Availability

The data presented in this study are available on request from the corresponding author. The data are not publicly available due to ethical considerations and participant confidentiality restrictions.

## Supplementary Materials

The following supporting information can be downloaded at: https://www.mdpi.com/article/10.3390/healthcare14172739/s1, COREQ (COnsolidated criteria for REporting Qualitative research) Checklist.

## Abbreviations

The following abbreviations are used in this manuscript:

RCC	Roles-Collaboration-Communication
DIPE-SA	Deprescribing Interprofessional Simulation Activity
