# Supplementary material for: Communication as Mediator: Interprofessional Deprescribing and the RCC (Roles–Collaboration–Communication) Framework
Source: Healthcare (Basel). 2026 Aug 27;14(17):2739. doi: 10.3390/healthcare14172739 (PMC13565896; doi:10.3390/healthcare14172739)
Supplement: Supplementary file 1 [file healthcare-14-02739-s001.zip › healthcare-4456957-supplementary.pdf]

Supplementary Materials. COREQ (COnsolidated criteria for REporting Qualitative research) Checklist.

Topic	Item No.	Guide Questions/Description	Response
**Domain 1: Research team and reflexivity**			
Personal characteristics			
Interviewer/facilitator	1	Which author/s conducted the interview or focus group?	Devin Scott & Alina Cernasev
Credentials	2	What were the researcher's credentials? E.g. PhD, MD	PhD, PhD & PharmD
Occupation	3	What was their occupation at the time of the study?	Assistant Professor and Instructional Consultant, Associate Professor
Gender	4	Was the researcher male or female?	Male, Female
Experience and training	5	What experience or training did the researcher have?	PhDs. Experienced qualitative researchers trained in qualitative data collection and analysis.
Relationship with participants			
Relationship established	6	Was a relationship established prior to study commencement?	Devin Scott and Alina Cernasev helped develop and conduct the Deprescribing Interprofessional Education Simulation Activity (DIPE-SA). Alina Cernasev teaches in the College of Pharmacy.
Participant knowledge of the interviewer	7	What did the participants know about the researcher? e.g. personal goals, reasons for doing the research	Devin Scott introduced himself and his role in the teaching and learning center. Alina Cernasev introduced herself as an instructor in the College of Pharmacy. Both interviewers shared their roles in developing and facilitating the DIPE-SA. Participants were informed of study goals during the reading of the consent statement.
Interviewer characteristics	8	What characteristics were reported about the inter viewer/facilitator? e.g. Bias, assumptions, reasons and interests in the research topic	None. Interest in the research topic was implied by development and facilitation of voluntary DIPE-SA.
**Domain 2: Study design**			
Theoretical framework			
Methodological orientation and Theory	9	What methodological orientation was stated to underpin the study? e.g. grounded theory, discourse analysis, ethnography, phenomenology, content analysis	Constructivist grounded theory approach.
Participant selection			
Sampling	10	How were participants selected? e.g. purposive, convenience, consecutive, snowball	Convenience Sample.

Method of approach	11	How were participants approached? e.g. face-to-face, telephone, mail, email	Email sent to participants in the DIPE-SA.
Sample size	12	How many participants were in the study?	19 participants from three colleges, in four focus groups.
Non-participation	13	How many people refused to participate or dropped out? Reasons?	Zero.
Setting			
Setting of data collection	14	Where was the data collected? e.g. home, clinic, workplace	Virtually, via online platform (Zoom®)
Presence of non-participants	15	Was anyone else present besides the participants and researchers?	No.
Description of sample	16	What are the important characteristics of the sample? e.g. demographic data, date	Table 1.
Data collection			
Interview guide	17	Were questions, prompts, guides provided by the authors? Was it pilot tested?	The interview guide was not pilot tested.<br>Representative sample questions:<br>1. Tell us about your experience going through the interprofessional deprescribing activity.<br>2. How did the activity affect your understanding of your role, and the roles of other healthcare professionals, in deprescribing?<br>3. What is your major takeaway from this interprofessional activity?
Repeat interviews	18	Were repeat inter views carried out? If yes, how many?	No.
Audio/visual recording	19	Did the research use audio or visual recording to collect the data?	Audio and visual data was collected. Transcription was completed with audio data only.
Field notes	20	Were field notes made during and/or after the interview or focus group?	Yes
Duration	21	What was the duration of the inter views or focus group?	60-75 minutes.
Data saturation	22	Was data saturation discussed?	Yes.
Transcripts returned	23	Were transcripts returned to participants for comment and/or correction?	No.

**Domain 3: analysis and findings**			
Data analysis			
Number of data coders	24	How many data coders coded the data?	Two. Alina Cernasev and Amy Hall. Devin Scott and Bruce Keisling joined as additional reviewers in cases of inconsistency.
Description of the coding tree	25	Did authors provide a description of the coding tree?	No. Major themes and subthemes are discussed.
Derivation of themes	26	Were themes identified in advance or derived from the data?	Themes were derived from data, in accordance with a constructivist grounded theory approach.
Software	27	What software, if applicable, was used to manage the data?	Dedoose® Software (Version 9.0.17, Los Angeles, California,

			USA)
Participant checking	28	Did participants provide feedback on the findings?	No.
Reporting			
Quotations presented	29	Were participant quotations presented to illustrate the themes/findings?<br>Was each quotation identified? e.g. participant number	Yes. Participant number, focus group number, and college are provided for each quotation.
Data and findings consistent	30	Was there consistency between the data presented and the findings?	Yes.
Clarity of major themes	31	Were major themes clearly presented in the findings?	Yes.
Clarity of minor themes	32	Is there a description of diverse cases or discussion of minor themes?	Minor themes are not discussed. However, two Communication subthemes are discussed.

Developed from: Tong A, Sainsbury P, Craig J. Consolidated criteria for reporting qualitative research (COREQ): a 32-item checklist for interviews and focus groups. Int J Qual Health Care 2007; 19:349–57.
